# Dentoskeletal and soft tissue changes in class II subdivision treatment with asymmetric extraction protocols

**DOI:** 10.1186/s40510-017-0193-x

**Published:** 2017-12-04

**Authors:** Guilherme Janson, Eduardo Beaton Lenza, Rodolfo Francisco, Aron Aliaga-Del Castillo, Daniela Garib, Marcos Augusto Lenza

**Affiliations:** 1 Department of Orthodontics, Bauru Dental School, University of São Paulo, Alameda Octávio Pinheiro Brisolla 9-75, Bauru, 17012-901 Brazil; 2 Department of Orthodontics, Dental School, Federal University of Goiás, Goiania, Brazil

**Keywords:** Class II subdivision, Premolar extractions, Asymmetric extractions, Cephalometrics

## Abstract

**Background:**

This study cephalometrically compared the dentoskeletal and soft tissue changes consequent to one and three-premolar extraction protocols of class II subdivision malocclusion treatment.

**Methods:**

A sample of 126 lateral cephalometric radiographs from 63 patients was selected and divided into two groups. Group 1 consisted of 31 type 1 class II subdivision malocclusion patients treated with asymmetric extractions of two maxillary premolars and one mandibular premolar on the class I side, with an initial mean age of 13.58 years. Group 2 consisted of 32 type 2 class II subdivision malocclusion patients treated with asymmetric extraction of one maxillary first premolar on the class II side, with an initial mean age of 13.98 years. *t* test was used for intergroup comparison at the pre- and posttreatment stages and to compare the treatment changes.

**Results:**

Group 1 had greater maxillomandibular sagittal discrepancy reduction and greater maxillary first molar extrusion. Group 2 had mandibular incisor labial inclination and protrusion, and group 1 had mandibular incisor lingual inclination and retraction. Maxillary molar asymmetry increased in group 2, while mandibular molar asymmetry increased in group 1.

**Conclusions:**

The treatment changes produced by these two class II subdivision protocols are different to adequately satisfy the different needs for types 1 and 2 class II subdivision malocclusions.

## Background

Two main types of class II subdivision malocclusions have been identified. Type 1 class II subdivision malocclusions are characterized by distal positioning of the mandibular first molar on the class II side, coincidence of the maxillary dental midline with the midfacial plane and deviation of the mandibular dental midline to the class II side, in a frontal clinical view [1–5]. Type 2 class II subdivision malocclusions are characterized by mesial positioning of the maxillary first molar on the class II side, deviation of the maxillary dental midline to the class I side and coincidence of the mandibular dental midline with the midfacial plane [4, 5]. There is also a third type, with combined characteristics of the first two types. Consequently, in this type, the maxillary midline is deviated to one side and the mandibular midline is deviated to the other [4, 5].

One of the treatment options for type 1 class II subdivision malocclusions consists in extractions of two maxillary premolars and one mandibular premolar on the class I side, as long as the facial profile and/or the amount of crowding allow extractions to be performed [1, 6–12]. For type 2 class II subdivision malocclusions, a treatment option may consist in extracting one maxillary premolar on the class II side [1, 4, 6, 8, 13, 14]. Therefore, it is speculated that the amount of dentoskeletal and soft tissue retraction is larger for the first treatment option. However, this has not been investigated.

When faced with the third type of class II subdivision malocclusion, the orthodontist may have to choose whether to treat it is as a type 1 or type 2 class II subdivision malocclusion, with the mentioned protocols. Therefore, knowing the differences in the dentoskeletal and soft tissue changes provided by both treatment protocols, it will be possible to select the best protocol to address the characteristics of the specific malocclusion.

Therefore, the objective of this work is to test the following null hypothesis: dentoskeletal and soft tissue changes are similar between type 1 class II subdivision malocclusions treated with two maxillary and one mandibular premolar extractions to type 2 class II subdivision malocclusions treated with one maxillary premolar extraction.

## Methods

This study was approved by the Ethics Committee of Bauru Dental School, University of São Paulo, Brazil. Sample size calculation, considering an 80% of test power at a significance level of 5%, with a minimum mean difference to be detected of 0.85 mm in 1-NB, with a standard deviation of 1.19 mm, revealed that 31 individuals in each of the two groups were the minimum amount necessary [9].

**Fig. 1:**
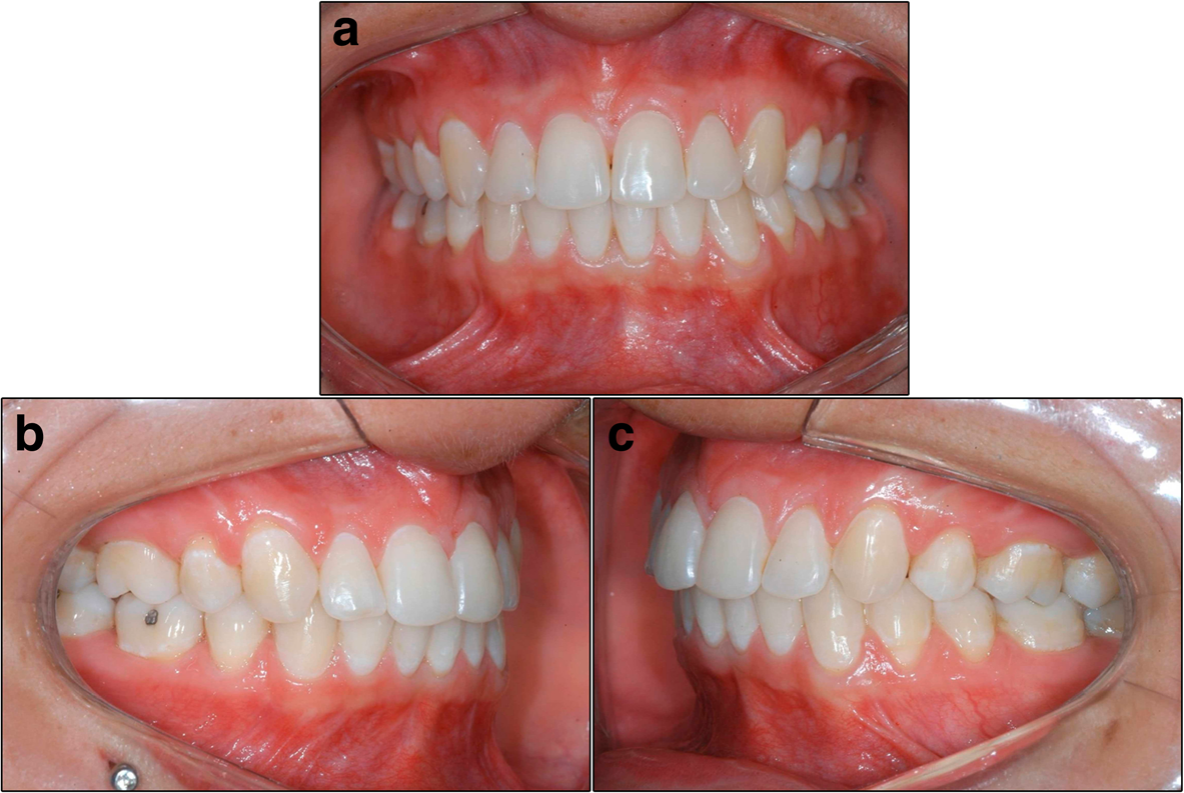
Group 1 patient – front (**a**), right side (**b**), left side (**c**)

**Fig. 2:**
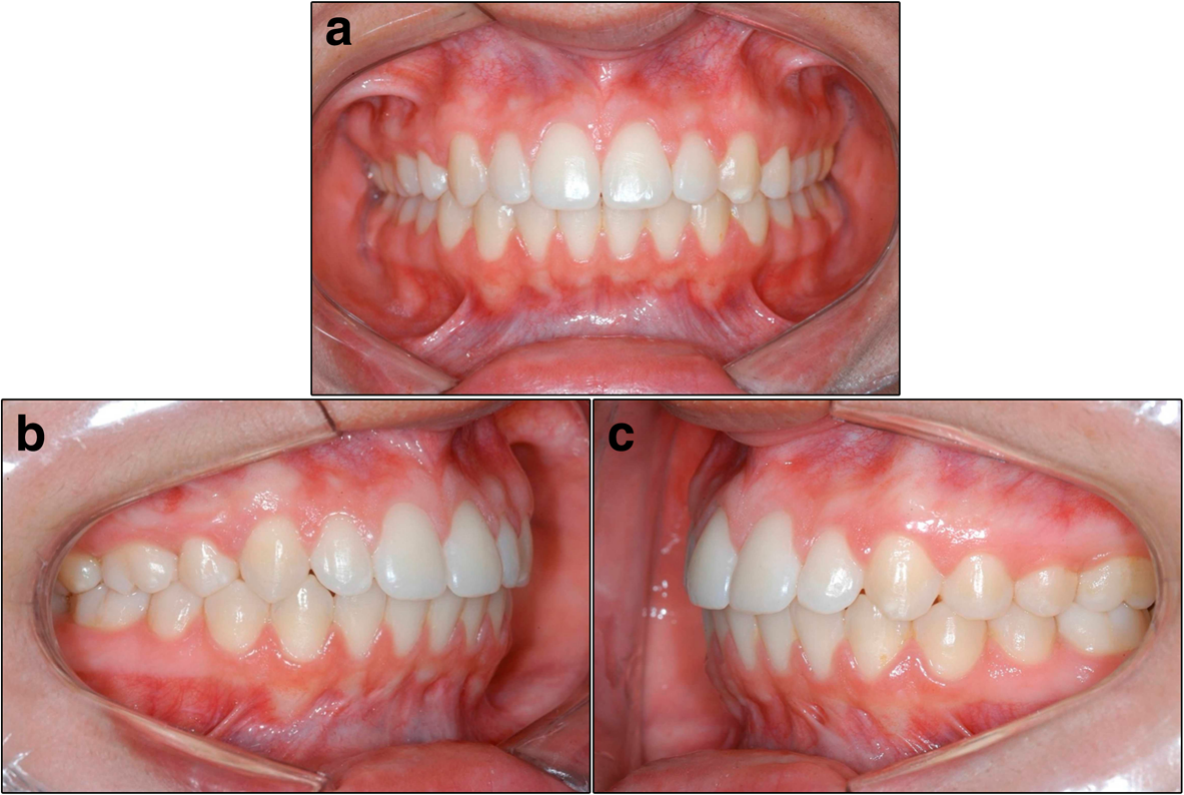
Group 2 patient – front (**a**), right side (**b**), left side (**c**)

Therefore, 63 patients, of White Mediterranean ancestry, initially presenting with class II subdivision malocclusions were retrospectively selected from the files of the Orthodontic Department at Bauru Dental School, University of São Paulo, Brazil. These patients were treated between 1998 and 2012 and were divided into two groups, according to their treatment approach. Group 1 consisted of 31 patients with type 1 class II subdivision malocclusions, with initial and final mean ages of 13.58 and 16.83 years, respectively, and a mean treatment time of 3.25 years, treated with asymmetric extractions of two maxillary premolars and one mandibular premolar on the class I side. Group 2 consisted of 32 patients with type 2 class II subdivision malocclusions, with initial and final mean ages of 13.98 and 16.90 years, respectively, and a mean treatment time of 2.92 years, treated with asymmetric extraction of one maxillary premolar on the class II side (Figs. 1 and 2).

The primary selection criterion was that patients presented a full class II molar relationship on one side and class I molar relationship on the opposite side. Additional selection criteria were: presence of all maxillary and mandibular permanent teeth up to the first molars, absence of supernumerary and impacted teeth, agenesis and anomalies of size and/or shape of the teeth, no facial trauma or medical history that could have altered the normal growth of their apical bases, no previous orthodontic treatment, initial and final records in satisfactory conditions, and good occlusal outcomes [2, 4].

All patients were treated with conventional edgewise or preadjusted fixed appliances (Roth prescription), both with 0.022 × 0.028 in. metalic brackets, by graduate students supervised by the same clinical instructor (GJ). Fixed or removable functional appliances were not used. The usual wire sequence began with 0.014 in. Niti archwires, followed by 0.016 in. Niti and 0.018, 0.020, and finally 0.018 × 0.025 in. stainless steel archwires. Thereafter, en-masse retraction of the anterior teeth was performed. Anchorage reinforcement with cervical extraoral headgear and lip bumpers (at the gingival margin of incisors) [9, 15] were used in all patients to maintain the original posterior teeth anteroposterior relationships. Class II elastics were used 18 h a day, for minor anteroposterior adjustment in the final stages, with 0.018 × 0.025 in. stainless steel archwires. Deep bites were usually corrected with accentuated and reversed curve of Spee on the archwires. As retention, the patients in both groups used Hawley plates on the maxillary arch and canine-to-canine bonded retainers.

**Fig. 3:**
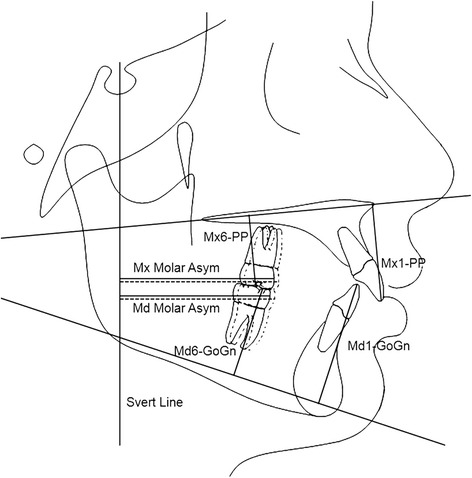
Skeletal and dental variables. Angular measurements: SNA; SNB; ANB; NAP; SN.GoGn; Mx1.NA; Md1.NB; IMPA. Linear measurements: Co-A; Co-Gn; LAFH; Overbite, Overjet, Mx1-NA; Md1-NB; Mx1-PP; Mx6-PP; Md1-GoGn; Md6-GoGn; Mx Molar Asymmetry; Md Molar Asymmetry

**Fig. 4:**
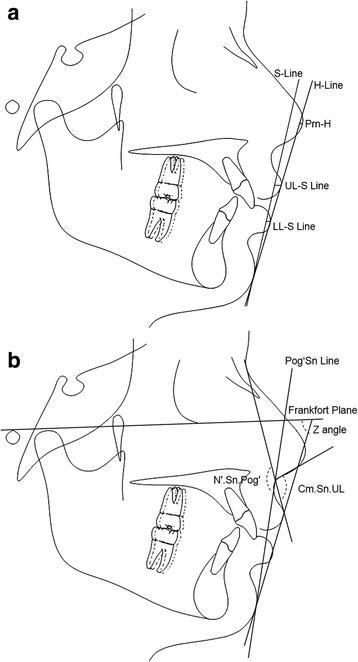
Soft tissue variables. Linear measurements (**a**): UL-S Line; Prn-H; LL-S Line. Angular measurements (**b**): Z Angle; N′.Sn.Pog’; Cm.Sn.UL

The pretreatment and posttreatment lateral cephalograms were obtained from each patient and scanned to allow the acquisition of images by Dolphin® Imaging 11.5 (Patterson Dental Supply, Inc., Chatsworth, CA). The magnification factors of the radiographic images that varied from 6 to 9.8% were corrected by the cephalometric software. Subsequently, 35 landmarks in the dentoskeletal facial structures and 17 landmarks in the soft tissue were marked (Figs. 3 and 4, Table 1).

**Table 1:** Skeletal, dental and soft tissue cephalometric variables

Maxillary skeletal components	
SNA	SN to NA angle
Co-A	Condylion to A-point distance
Mandibular skeletal components	
SNB	SN to NB angle
Co-Gn	Condylion to gnathion distance
Maxillomandibular relationship	
ANB	NA to NB angle
NAP	Angle between lines NA and AP
Growth pattern	
SN.GoGn	SN to GoGn angle
LAFH	ANS, anterior nasal spine to menton distance
Maxillary dentoalveolar components	
Mx1.NA	Maxillary incisor long axis to NA angle
Mx1-NA	Distance between most anterior point of crown of maxillary incisor and NA line
Mx1-PP	Perpendicular distance between the incisal edge of the maxillary incisor and the palatal plane
Mx6-PP	Perpendicular distance between the maxillary first molar mesial and distal cusps midpoint and the palatal plane
Mx Molar asymmetry	Difference in the distance between most mesial points of right and left maxillary first molars, perpendicular to Svert Line (Mx6-Svert)
Mandibular dentoalveolar components	
Md1.NB	Mandibular incisor long axis to NB angle
Md1-NB	Distance between most anterior point of crown of mandibular incisor and NB line
IMPA	Incisor mandibular plane angle
Md1-GoGn	Perpendicular distance between the incisal edge of the mandibular incisor and the mandibular plane
Md6-GoGn	Perpendicular distance between the mesiobuccal cusp tip of the mandibular first molar and the mandibular plane
Md Molar asymmetry	Difference in the distance between most mesial points of right and left maxillary first molars, perpendicular to Svert Line (Md6-Svert)
Dental relationship	
Overjet	Distance between incisal edges of maxillary and mandibular central incisors, parallel to occlusal plane
Overbite	Distance between incisal edges of maxillary and mandibular central incisors, perpendicular to occlusal plane
Upper lip	
UL-S line	Distance from the upper lip to Steiner’s S line (line from Pg’ to Cl)
Prn-H	Distance between H line and the most anterior point on the nose
Lower lip	
LL-S line	Distance from the lower lip to Steiner’s S line
Facial convexity and nasolabial angle	
Z angle	Angle formed by the intersection of Frankfort horizontal plane and a line connecting the soft-tissue chin (Pg’) and the most protrusive lip point
N’.Sn.Pog’	Facial convexity
Cm.Sn.UL	Nasolabial angle

For dental asymmetry assessment, two digital tracings were performed for each patient at T1 and T2. In the first tracing, linear measurements were made from the mesial point of the most mesial maxillary and mandibular molar, perpendicularly, to the Svert Line (6-Svert). In the second tracing, linear measurements were made from the mesial point of the most distal maxillary and mandibular molar, perpendicularly, to the same vertical reference line (6-Svert). Asymmetry was calculated as the difference between the most mesial and distal molars for the maxillary and mandibular molars [2, 9].

The Little irregularity index [16] was used to calculate crowding at the pretreatment stage. This index was originally used to evaluate anterior mandibular dental crowding, and has been adapted to also quantify maxillary anterior crowding [17].

### Error study

Twenty-two lateral cephalograms were randomly selected and remeasured by the same examiner (EBL), after a 30-day interval. Random errors were calculated according to Dahlberg’s formula (*S*e^2^ = ∑*d* ^2^/2*n*) [18], where *S* ^*2*^ is the error variance and *d* is the difference between 2 determinations of the same variable and the systematic errors were evaluated with dependent *t* tests, at *P* < 0.05 [19].

### Statistical analyses

The means and standard deviations (SD) for each variable were calculated for both groups. Kolmogorov-Smirnov tests were applied to verify normal distribution of the variables. The results of the tests were nonsignificant for all variables. Therefore, intergroup comparability was evaluated with *t* tests regarding the initial and final ages, treatment time and the cephalometric characteristics at the pretreatment stage. Chi-square test was used to compare sex distribution in the groups.

*t* tests were also used to compare the treatment changes and the cephalometric status at the posttreatment stage. All tests were performed with Statistica software (Version 7, StatSoft Inc., Tulsa, OK, USA), at *P* < .05.

## Results

The random errors ranged from 0.44 (overbite) to 1.38 mm (CoGn) and from 0.77 (SND) to 1.45° (Cm.Sn.Ls). Only two variables (LAFH and Z angle) presented significant systematic errors. The groups were comparable regarding the initial age, treatment time, sex distribution, and initial occlusal characteristics (Table 2). At the pretreatment stage, group 2 had significantly smaller maxillary incisor dentoalveolar development, greater maxillary molar asymmetry, smaller mandibular molar asymmetry, greater nasal prominence, and smaller lower lip protrusion (Table 3).

**Table 2:** Intergroup baseline comparability (*t* and Chi-square tests)

	Group 1—3 extractions (*n* = 31)		Group 2—1 extraction (*n* = 32)		
Variables	Mean	S.D.	Mean	S.D.	*P*
Initial age	13.58	2.26	13.98	1.66	0.425^a^
Final age	16.83	2.39	16.90	1.85	0.888^a^
Treatment time	3.25	1.02	2.92	1.02	0.210^a^
Sex					
Male	13		12		
Female	18		20		0.719^b^
Maxillary Little Irregularity Index	5.33	4.02	4.22	2.74	0.202^a^
Mandibular Little Irregularity Index	2.52	1.52	2.43	1.74	0.824^a^

^a^ *t* test

^b^Chi-square test

**Table 3:** Intergroup comparison at the pretreatment stage (*t* tests)

	Group 1—3 extractions (*n* = 31)		Group 2—1 extraction (*n* = 32)		
Variables	Mean	S.D.	Mean	S.D.	*P*
Maxillary component					
SNA (°)	84.10	4.10	83.28	4.04	0.429
Co-A (mm)	82.65	3.81	83.41	3.53	0.415
Mandibular component					
SNB (°)	79.32	3.98	79.26	3.06	0.946
Co-Gn (mm)	110.95	4.59	111.96	5.89	0.451
Maxillomandibular relationship					
ANB (°)	4.77	1.75	4.02	2.19	0.137
NAP (°)	171.65	4.39	172.88	4.11	0.254
Growth pattern					
SN.GoGn (°)	29.19	3.96	27.86	4.36	0.210
LAFH (mm)	63.43	4.33	62.32	4.41	0.316
Maxillary dentoalveolar component					
Mx1.NA (°)	24.45	5.49	25.75	7.16	0.423
Mx1-NA (mm)	5.53	2.44	5.50	3.86	0.971
Mx1-PP (mm)	28.40	2.23	26.81	2.95	0.018^a^
Mx6-PP (mm)	20.35	2.36	19.43	1.98	0.098
Mx Molar asymmetry (mm)	1.99	0.33	2.19	0.39	0.025^a^
Mandibular dentoalveolar component					
Md1.NB (°)	28.82	5.01	27.85	6.02	0.489
Md1-NB (mm)	5.87	1.41	5.94	2.10	0.872
IMPA (°)	97.56	5.83	99.22	7.93	0.350
Md1-GoGn (mm)	35.97	2.29	35.46	2.86	0.442
Md6-GoGn (mm)	26.83	2.19	27.32	2.33	0.387
Md Molar asymmetry (mm)	2.09	0.46	1.79	0.57	0.025^a^
Dental relationship					
Overjet (mm)	4.88	1.80	4.96	1.78	0.856
Overbite (mm)	1.89	2.31	1.97	1.10	0.863
Upper lip					
UL-S line (mm)	2.41	2.42	1.41	1.98	0.077
Prn-H (mm)	0.91	4.56	3.31	4.00	0.030^a^
Lower lip					
LL-S line (mm)	3.50	3.04	1.95	2.46	0.029^a^
Facial convexity and nasolabial angle					
Z Angle (°)	75.85	5.51	73.92	6.61	0.214
N’.Sn.Pog’(°)	156.08	5.13	157.63	6.07	0.276
Cm.Sn.UL (°)	105.07	8.93	101.23	8.26	0.081

^a^Statistically significant at *P* < 0.05

During treatment, there was significantly greater maxillomandibular relationship improvement, decrease in facial convexity, and increase in molar dentoalveolar height in group 1 (Table 4). Group 2 presented significantly greater increase in maxillary molar asymmetry. There was significant differences regarding mandibular incisor behavior. While group 2 had labial tipping and protrusion of the incisors, group 1 had lingual tipping and retrusion. Group 1 had greater increase in mandibular molar asymmetry.

**Table 4:** Intergroup comparison of treatment changes (*t* tests)

	Group 1—3 extractions (*n* = 31)		Group 2—1 extraction (*n* = 32)		
Variables	Mean	S.D.	Mean	S.D.	*P*
Maxillary component					
SNA (°)	− 1.73	3.17	− 0.58	2.79	0.131
Co-A (mm)	− 0.95	4.12	− 0.29	2.00	0.416
Mandibular component					
SNB (°)	0.49	2.27	− 0.02	2.27	0.376
Co-Gn (mm)	3.55	5.14	1.98	3.27	0.153
Maxillomandibular relationship					
ANB (°)	− 2.21	2.19	− 0.56	1.87	0.002^a^
NAP (°)	2.95	4.44	0.49	2.79	0.010^a^
Growth pattern					
SN.GoGn (°)	− 0.58	2.89	− 1.18	3.12	0.434
LAFH (mm)	2.45	2.56	1.10	3.51	0.088
Maxillary dentoalveolar component					
Mx1.NA (°)	− 1.11	6.38	1.91	7.80	0.098
Mx1-NA (mm)	− 0.68	3.09	− 0.38	3.27	0.716
Mx1-PP (mm)	− 0.66	1.24	− 0.43	1.74	0.533
Mx6-PP (mm)	1.96	2.10	0.78	1.98	0.024^a^
Mx Molar asymmetry (mm)	− 0.16	0.43	0.75	1.13	0.000^a^
Mandibular dentoalveolar component					
Md1.NB (°)	− 1.03	5.08	3.09	4.59	0.001^a^
Md1-NB (mm)	− 0.47	0.96	0.86	1.34	0.000^a^
IMPA (°)	− 0.67	5.58	3.13	5.62	0.009^a^
Md1-GoGn (mm)	0.98	2.05	0.49	1.86	0.325
Md6-GoGn (mm)	2.12	1.67	1.40	1.63	0.087
Md Molar asymmetry (mm)	1.06	0.57	0.09	0.72	0.000^a^
Dental relationship					
Overjet (mm)	− 2.23	1.72	− 2.03	1.87	0.648
Overbite (mm)	− 0.88	2.27	− 1.16	1.31	0.551
Upper lip					
UL-S Line (mm)	− 1.85	2.34	− 1.28	1.44	0.241
Prn-H (mm)	3.80	4.43	2.65	2.88	0.223
Lower lip					
LL-S Line (mm)	− 1.68	2.27	− 0.88	1.47	0.101
Facial convexity and nasolabial angle					
Z Angle (°)	2.15	4.72	0.68	5.41	0.254
N’.Sn.Pog’(°)	1.41	4.68	0.03	3.89	0.207
Cm.Sn.UL (°)	1.71	7.73	1.69	9.22	0.992

^a^Statistically significant at *P* < 0.05

At the posttreatment stage, group 1 demonstrated significantly greater LAFH, smaller labial tipping, and greater dentoalveolar height of the maxillary incisors (Table 5). Group 1 also presented greater maxillary molar dentoalveolar height and smaller maxillary molar asymmetry. Group 2 presented greater labial tipping and protrusion of the mandibular incisors, smaller mandibular molar asymmetry, and greater facial convexity.

**Table 5:** Intergroup comparison at the posttreatment stage (*t* tests)

	Group 1—3 extractions (*n* = 31)		Group 2—1 extraction (*n* = 32)		
Variables	Mean	S.D.	Mean	S.D.	*P*
Maxillary component					
SNA (°)	82.37	4.75	82.70	4.65	0.778
Co-A (mm)	81.77	4.00	83.12	3.46	0.156
Mandibular component					
SNB (°)	79.81	4.22	79.24	2.55	0.518
Co-Gn (mm)	114.50	6.30	113.95	5.36	0.708
Maxillomandibular relationship					
ANB (°)	2.56	2.44	3.46	2.83	0.183
NAP (°)	174.60	3.95	173.38	3.86	0.218
Growth pattern					
SN.GoGn (°)	28.61	5.45	26.68	4.80	0.140
LAFH (mm)	65.88	4.76	63.42	4.77	0.044^a^
Maxillary dentoalveolar component					
Mx1.NA (°)	23.34	5.21	27.66	8.11	0.014^a^
Mx1-NA (mm)	4.85	2.42	5.12	4.13	0.758
Mx1-PP (mm)	27.74	2.27	26.38	2.81	0.039^a^
Mx6-PP (mm)	22.31	2.21	20.20	2.19	0.000^a^
Mx Molar asymmetry (mm)	1.83	0.42	2.94	1.07	0.000^a^
Mandibular dentoalveolar component					
Md1.NB (°)	27.79	5.18	30.94	5.13	0.018^a^
Md1-NB (mm)	5.42	1.39	6.80	2.00	0.002^a^
IMPA (°)	96.89	6.49	102.34	7.39	0.002^a^
Md1-GoGn (mm)	36.95	2.94	35.95	2.89	0.180
Md6-GoGn (mm)	28.95	2.28	28.72	2.58	0.713
Md Molar asymmetry (mm)	3.15	0.58	1.88	0.54	0.000^a^
Dental relationship					
Overjet (mm)	2.65	0.68	2.94	0.73	0.109
Overbite (mm)	1.01	0.74	0.81	0.95	0.353
Upper lip					
UL-S Line (mm)	0.56	2.67	0.14	2.55	0.524
Prn-H (mm)	4.72	5.07	5.96	4.64	0.312
Lower lip					
LL-S Line (mm)	1.81	2.86	1.07	2.48	0.272
Facial convexity and nasolabial angle					
Z Angle (°)	77.99	6.95	74.59	4.46	0.023^a^
N’.Sn.Pog’(°)	157.49	5.94	157.67	5.90	0.905
Cm.Sn.UL (°)	106.79	9.51	102.93	10.55	0.132

^a^Statistically significant at *P* < 0.05

## Discussion

### Groups’ comparability

Only class II subdivision malocclusion patients, with complete class II on one side and class I on the other, independently of the associated cephalometric factors were included [7]. Group 1 had significantly greater maxillary incisor dentoalveolar height, smaller maxillary molar asymmetry, and greater mandibular molar asymmetry (Table 3). The greater maxillary incisor dentoalveolar height suggests a slightly more accentuated vertical growth pattern and the differences in molar asymmetry are characteristics of the two types of subdivision malocclusions [4–6, 11, 20]. Group 1 had also greater lower lip protrusion which might also have contributed for the extraction treatment in this group. However, these slight differences should not interfere with the comparison.

### Treatment changes and posttreatment status

The greater reduction in maxillomandibular relationship in group 1 may be consequent to the greater number of extractions in the maxillary arch, and increased need for class II elastics or headgear, to reinforce anchorage, that consequently produced a non-significantly greater maxillary retraction [21–23], Table 4. Group 1 also had a non-significantly greater increase in mandibular growth. These non-significant greater changes in group 1, when associated, might have contributed for a significantly greater reduction in maxillomandibular relationship than group 2. Additionally, these associated greater non-significant changes might have also contributed for a greater reduction in skeletal facial convexity in group 1. However, these greater treatment changes of group 1 were not enough to produce intergroup differences at the posttreatment stage (Table 5).

Despite there were no significant intergroup differences in maxillary incisor treatment changes, they were significantly more palatally tipped and had greater dentoalveolar height in group 1, at the posttreatment stage (Tables 4 and 5). The greater palatal inclination was probably consequent to the non-significant greater palatal tipping that occurred during treatment in this group because of incisor retraction to close the two maxillary premolar extraction spaces. Group 1 already had greater dentoalveolar height at the pretreatment stage and because the vertical treatment changes were similar in the groups, the initial intergroup pretreatment difference was maintained.

The greater increase in molar dentoalveolar height in group 1 was probably due to greater need of intermaxillary elastics and/or headgear use, associated with the slightly greater vertical growth pattern of this group [7, 9, 24], Table 4. The three premolar extraction protocol performed in group 1 requires greater amount of anchorage reinforcement during anterior retraction, compared to the one premolar extraction protocol performed in group 2. Therefore, this may have contributed to more extrusion in group 1. Consequently to this, LAFH and maxillary molar dentoalveolar height were significantly greater in group 1, at the posttreatment stage, which usually occurs [9, 24], Table 5.

Because group 2 had only one maxillary premolar extraction, it is quite obvious that maxillary molar asymmetry had greater increase in this group than in group 1, which increased even more the significant initial intergroup difference in molar asymmetry (Tables 4 and 5). Nevertheless, this amount of asymmetry between the maxillary molars in group 2 is not clinically relevant and does not bring any decrease in smile attractiveness [13, 25, 26].

The significant intergroup differences in mandibular incisor behavior was consequent to the non-extraction treatment in group 2 and one premolar extraction treatment in group 1 (Table 4). Therefore, there was mandibular incisor labial inclination and protrusion resulting from correction of the anterior crowding and leveling of the curve of Spee without mandibular premolar extraction in group 2 [11, 27, 28]. In group 1, there was retraction and lingual inclination of the mandibular incisors as a result of space closure of one mandibular premolar extraction [13, 29]. Consequent to these different changes, the mandibular incisors of group 1 were significantly more lingually tipped and retruded than those of group 2 at the posttreatment stage (Table 5).

Similarly to the maxillary molars because group 1 had only one mandibular premolar extraction, it is quite obvious that mandibular molar asymmetry had greater increase in this group than in group 2, which increased even more the significant initial intergroup difference in molar asymmetry, at the posttreatment stage (Tables 4 and 5).

Group 1 presented a significantly smaller facial convexity at the posttreatment stage, probably because of the non-significanly greater upper lip retrusion, with treatment, than group 2 [21, 29–32].

The two treatment protocols for class II subdivision malocclusions produced significantly different changes in certain dentoalveolar variables. This is reasonable because each of these protocols are indicated for different types of class II subdivision malocclusions. For type 1, three premolar extractions are indicated [1, 6–9, 11–13]. This is the case when maxillary incisor protrusion or crowding is accentuated and there is also some mandibular crowding or incisor protrusion [7, 9, 11, 12, 33]. Therefore, this protocol will solve these problems [8, 13, 24]. For type 2, there is usually less maxillary incisor protrusion and there is no mandibular crowding, with the incisors labiolingually well positioned. Therefore, the treatment changes will be favorable according to each malocclusion need.

## Conclusions

The null hypothesis was rejected because the treatment changes had the following differences:

1. There was greater maxillomandibular sagittal discrepancy reduction in group 1;
2. There was greater maxillary first molar extrusion in group 1;
3. Maxillary molars asymmetry increased in group 2 and decreased in group 1.
4. There was greater mandibular incisor labial inclination and protrusion in group 2, and mandibular incisor lingual inclination and retrusion in group 1;
5. There was greater increase of mandibular molar asymmetry in group 1.
